# Genetic diversity and antifungal susceptibilities of environmental *Cryptococcus neoformans* and *Cryptococcus gattii* species complexes

**DOI:** 10.1186/s43008-024-00153-w

**Published:** 2024-07-25

**Authors:** Mohamed Taha, Yasmine H. Tartor, Rana M Abd Elaziz, Ibrahim Elsohaby

**Affiliations:** 1 Department of Microbiology, Faculty of Veterinary Medicine, Zagazig University, Zagazig, 44511 Egypt; 2 Cairo International Airport Veterinary Quarantine, General Organization for Veterinary Services, Ministry of Agriculture, Cairo, Egypt; 3 Department of Infectious Diseases and Public Health, Jockey Club College of Veterinary Medicine and Life Sciences, City University of Hong Kong, Hong Kong SAR, China; 4 Centre for Applied One Health Research and Policy Advice (OHRP), City University of Hong Kong, Hong Kong, Hong Kong SAR, China; 5 Department of Animal Medicine, Faculty of Veterinary Medicine, Zagazig University, Zagazig, 44511 Egypt

**Keywords:** *Cryptococcus neoformans*, *Cryptococcus gattii*, Environmental isolates, Antifungal susceptibility, Minimum inhibitory concentration, Antifungal combination, Genotyping

## Abstract

Cryptococcosis is an opportunistic systemic mycosis caused by *Cryptococcus neoformans* and *C. gattii* species complexes and is of increasing global importance. Maintaining continued surveillance of the antifungal susceptibility of environmental *C. neoformans* and *C. gattii* isolates is desirable for better managing cryptococcosis by identifying resistant isolates and revealing the emergence of intrinsically resistant species. Relevant research data from Egypt are scarce. Thus, this study aimed to report the genetic diversity of *C. neoformans* and *C. gattii* species complexes originating from different environmental sources in Egypt, antifungal susceptibility profiles, antifungal combinations, and correlations of susceptibility with genotypes. A total of 400 environmental samples were collected, 220 from birds and 180 from trees. *Cryptococcus* spp. were found in 58 (14.5%) of the samples, 44 (75.9%) of the isolates were recovered from birds and 14 (24.1%) from trees. These isolates were genotyped using M13 polymerase chain reaction-fingerprinting and *URA*5 gene restriction fragment length polymorphism analysis. Of the 31 *C. neoformans* isolates, 24 (77.4%), 6 (19.4%) and one (4.4%) belonged to VNI, VNII, and VNIII genotypes, respectively. The 27 *C. gattii* isolates belonged to VGI (70.4%), VGII (18.5%), and VGIII (11.1%) genotypes. Non-wild type *C. neoformans* and *C. gattii* isolates that may have acquired resistance to azoles, amphotericin B (AMB), and terbinafine (TRB) were observed. *C. gattii* VGIII was less susceptible to fluconazole (FCZ) and itraconazole (ITZ) than VGI and VGII. *C. neoformans* isolates showed higher minimum inhibitory concentrations (MICs) to FCZ, ITZ, and voriconazole (VRZ) than those of *C. gattii* VGI and VGII. Significant (*P* < 0.001) correlations were found between the MICs of VRZ and ITZ (*r* = 0.64) in both *C*. *neoformans* and *C. gattii* isolates, FCZ and TRB in *C. neoformans* isolates, and FCZ and TRB (*r* = 0.52) in *C. gattii* isolates.

There is no significant differences in the MICs of TRB in combination with FCZ (*P* = 0.064) or in combination with AMB (*P* = 0.543) and that of TRB alone against *C. gattii* genotypes. By calculating the fractional inhibitory concentration (FIC) index, the combination of FCZ + AMB was synergistic against all tested genotypes. These findings expand our knowledge of ecological niches, genetic diversity, and resistance traits of *C. neoformans* and *C. gattii* genotypes in Egypt. Further investigations into how they are related to clinical isolates in the region are warranted.

**Supplementary Information:**

The online version contains supplementary material available at 10.1186/s43008-024-00153-w.

## Introduction

Cryptococcosis is a systemic life-threatening opportunistic fungal disease that affects internal organs and skin in both humans and animals, particularly in immunocompromised hosts (Alves et al. 2016). *Cryptococcus* infection is acquired by inhalation of basidiospores and/or desiccated yeast cells from environmental sources, including pigeon (*Columba livia*) excreta, plant debris, and decayed wood. Initial pulmonary infection can occur by penetrating the lung, causing acute pneumonia, with subsequent dissemination to the brain (manifesting as highly fatal meningitis) and other organs (Velagapudi et al. 2009; Harris et al. 2012; Walsh et al. 2019).

Among the numerous *Cryptococcus* species, *Cryptococcus neoformans* and *C. gattii* species complexes are considered the primary causative agents of cryptococcosis which has a global distribution (Kwon-Chung et al. 2014; Fang et al. 2015). Although most infected patients with disseminated cryptococcosis are immunocompromised, *C. neoformans* can cause disease in apparently healthy hosts. In contrast, a significantly higher proportion of immunocompetent patients are affected by *C. gattii* infections (Kwon-Chung et al. 2014).

Cryptococcosis is an important disease that affects a wide range of animals worldwide, including cattle, sheep, goats, horses, cats, dogs, and birds. In domestic animals, it is commonly associated with mastitis in cattle, sheep, and goats, as well as endometritis and placentitis in mares (Refai et al. 2017). However, a variety of other animals, including terrestrial wildlife species and marine mammals, can also show clinical signs, pathological findings, and potential underlying causes of cryptococcosis. This may be due to the animal's behaviour and environmental exposures. For example, Koalas primarily exhibit pulmonary lesions caused by *C*. *gattii* species (VGI and VGII) as a result of their behaviour and environmental exposure to *Eucalyptus* trees (Danesi et al. 2021).

The most used approaches for genotyping are PCR fingerprinting, restricted fragment length polymorphism (PCR–RFLP), amplified fragment length polymorphism (AFLP), multilocus sequence typing (MLST), and matrix-assisted laser desorption ionization time of flight mass spectrometry (MALDI-TOF MS) analysis. These methods have demonstrated the ability to distinguish between molecular types of *Cryptococcus* genus in both clinical and environmental isolates (Meyer et al. 2009; Hagen et al. 2015; Chen et al. 2018).

DNA typing techniques, using microsatellite-specific primer [GACA]4, divided *C. neoformans* into four major molecular types (AFLP1/VNI, AFLP1A/VNB/VNII, AFLP1B/ VNII, AFLP3/VNIII, and AFLP2/VNIV). These types are characterized by differences in pathogenicity, geographical distribution, and susceptibility to antifungal treatments (Pini et al. 2017). Environmental isolates of C. *neoformans* recovered from pigeon droppings in East China were genetically more divers than clinical isolates (Chen et al. 2021). In Korea, a strong linkage was observed between clinical and environmental *Cryptococcus* isolates (Park et al. 2015). Additionally, environmental *Cryptococcus* isolates VNI and VGII were similar to those causing human infection in Brazil (Alves et al. 2016) and Latin America (Firacative et al. 2021). *C. gattii* VGII was also found to be responsible for animal infections in Latin America (Firacative et al. 2021). On Vancouver Island (Canada), *C. gattii* VGII was identified as the most prevalent molecular type in human, animal infections, and environmental samples (Kidd et al. 2004).

Long-term usage of therapeutic and/or prophylactic antifungal drugs has led to the emergence of resistance in *C. neoformans* and *C. gattii* species (Arechavala et al. 2009). Thus, antifungal susceptibility is important in epidemiological investigations for tracking susceptibility profiles and drug resistance (Taha et al. 2020). Differences in the antifungal susceptibility of *C. neoformans* and *C. gattii* species complexes have been reported to differ according to genotype and geographic origin of isolates (Andrade-Silva et al. 2023; Chong et al. 2010; Hagen et al. 2010; Iqbal et al. 2010; Trilles et al. 2012). For instance, *C*. *gattii* VGII isolates from Australia, Canada, and USA have been reported to be less susceptible to azoles than other molecular types (Chong et al. 2010; Hagen et al. 2010). Environmental *C. neoformans* isolates recovered from pigeon droppings in China were fluconazole-resistant, and the rate of itraconazole resistance was higher than that of clinical isolates (Chen et al. 2021).

Taken together, this study was the first to investigate the genotypes and susceptibility profiles of environmental *C. neoformans* and *C. gattii* species complexes from different localities in Egypt.

## Materials and methods

### Environmental samples collection

A total of 400 samples were collected between October and December 2019. The samples comprised of 220 bird droppings and 180 samples were obtained from the leaves and woody trunks of *Eucalyptus* and olive trees. Specifically, 120 pigeon droppings were obtained from pigeon nests in pet shops, houses, and towers in Zagazig city and Miniaelqmh city, Sharkia Governorate. Additionally, captive bird (canary) droppings (50 samples) were collected from pet shops and houses in Zagazig city. Furthermore, 50 zoo bird droppings were collected from four large cages in Giza Zoo, Egypt. All samples were collected using a clean spatula and stored in clean plastic bags, which were then kept refrigerated until examination. The *Eucalyptus* tree samples (*n* = 130) were collected from various locations in Cairo, Sharkia, and Qalubiya Governorates. The olive tree samples (*n* = 50) were collected from private houses and farms located in Cairo, Sharkia, and the Cairo-Alexandria Desert Road.

### Isolation and identification of yeast isolates

To prepare each bird dropping for testing, a suspension was prepared by adding 9 mL of sterile saline solution to 1 g of the sample. The resulting mixture was centrifuged at 3000 rpm for 5 min. The sediment was subsequently inoculated onto two Petri plates of Sabouraud dextrose agar (SDA) medium supplemented with chloramphenicol (Himedia, India). The plates were then incubated at 25ºC and 37ºC for 72 h (Li et al. 2000). The preparation of the *Eucalyptus* and olive tree samples were prepared as previously described (Kidd et al. 2007). Briefly, five grams of each specimen was suspended in 25 mL of sterile saline solution. The mixture was then vortexed and allowed to settle for approximately20 min. A loopful of each sample was inoculated onto two SDA plates and incubated at 25ºC and 37ºC for 72 h.

Creamy, mucoid yeast isolates were picked from the primary culture onto SDA slopes using a sterile loop. The identification process involved both phenotypic and molecular methods. *Cryptococcus* isolates were identified based on macromorphology, micromorphology, and physiological characters such as urea hydrolysis and changing color on *Cryptococcus* differential agar medium (Himedia, India) (Granados and Castañeda 2005; Singh et al. 2013).

### Molecular identification and genotyping of* Cryptococcus* species isolates

#### Multiplex PCR for *Cryptococcus* species identification

DNA was extracted using a QIAamp DNA Mini Kit (catalog no. 51304; Sigma, USA) following the manufacturer’s instructions. The amplification reaction (50 μL per sample) included 25 μL of EmeraldAmp GT PCR master-mix (Code No. RR310A, Takara, USA), 1 μL (20 pmol) of each primer targeting the aminotransferase gene of *C. neoformans* (CNa-70S forward 5ˊATTGCGTCCACCAAGGAGCTC 3ˊ and CNa-70A reverse 5ˊATTGCGTCCATGTTACGTGGC 3ˊ) and the polymerase gene of *C. gattii* (CNb-49S forward 5ˊATTGCGTCCAAGGTGTTGTTG 3ˊ and CNb-49A reverse 5ˊATTGCGTCCATCCAACCGTTATC 3ˊ targeting), 6 μL of template DNA, and nuclease-free water up to 50 μL. The amplification parameters consisted of primary denaturation at 94 °C for 8 min; 35 cycles of secondary denaturation at 94 °C for 1 min, annealing at 56 °C for 1 min, and extension at 72 °C for 2 min; and a final extension at 72 °C for 8 min (Leal et al. 2008).

#### Fingerprinting PCR

PCR was performed using the minisatellite-specific core sequence of the wild-type phage M13 primer (5ˊGAGGGTGGCGGTTCT 3ˊ) in a total volume of 50 µL for 35 cycles of denaturation at 94 °C for 20 s, annealing at 50 °C for 1 min, and extensions at 72 °C for 20 s, followed by a final extension cycle for 6 min at 72 °C (Meyer et al. 2003). PCR fingerprinting types (VNI-VNII-VNIII and VGI-VGII-VGIII) were assigned according to the major bands that were typical for that pattern. Bands were included in the analysis regardless of their intensity if they were visible.

#### URA5 gene RFLP analysis

PCR amplification of the orotidine monophosphate pyrophosphorylase *(URA5)* gene was performed using the URA5 (5ˊATGTCCTCCCAAGCCCTCGACTCCG 3ˊ) and SJ01 (5ˊTTAAGACCTCTGAACACCGTACTC 3ˊ) primers. Thirty-five cycles of initial denaturation at 94 °C for 2 min, second denaturation at 94 °C for 45 s, annealing at 61 °C for 1 min, and extension at 72°Cfor 2 min, followed by a final extension cycle for 10 min at 72 °C (Meyer et al. 2003). Amplification products were mixed with one fifth volume of loading buffer, 15 µL of PCR products was double digested with *Sau*96I (10 U/µL) and *Hha*I (20 U/µl) for 3 h. RFLP patterns were assigned by comparing them with the patterns obtained from the standard strains (VNI-VNIII and VGI-VGIII). The molecular types for each isolate were determined by comparing the obtained M13 PCR fingerprint profiles and *URA5* RFLP patterns with the respective standard patterns for each molecular type.

### Antifungal susceptibility testing

The minimal inhibitory concentrations (MICs) of six antifungal drugs, namely amphotericin (AMB; Sigma–Aldrich, Basingstoke, UK), fluconazole (FCZ; Pfizer, Sandwich, UK), itraconazole (ITZ; Janssen, Beerse, Belgium), voriconazole (VRZ; Pfizer, Sandwich, UK), ketoconazole (KETO; Sigma–Aldrich, Basingstoke, UK), and terbinafine (TRB, Novartis, Switzerland), were determined using the broth microdilution method following the Clinical and Laboratory Standards Institute M57S-Ed4 guidelines (Clinical and Laboratory Standards Institute (CLSI) 2022). The inoculum concentrations ranged from 0.5 × 10^3^ to 2.5 × 10^3^ CFU/mL and were incubated at 35 °C for 72 h. TRB, AMB, VRZ, and ITZ were dissolved in dimethyl sulfoxide (DMSO; Sigma-Aldrich, Germany), while FCZ was dissolved in water then diluted in RPMI 1640 (Sigma-Aldrich) (range AMB: 0.03–16 µg/mL, FCZ: 0.125–64 µg/mL, ITZ and VRZ 0.015–8 µg/mL, KTZ: 0.03–64 µg/mL, and TRB: 0.06–8 µg/mL). Each test included a positive control (drug-free growth control) and a negative control (RPMI 1640 medium). *Candida parapsilosis* ATCC 22019 was included in the test as a quality control (QC) strain. The test was performed twice to confirm the results and MICs were determined visually.

For AMB, the MICs were defined as the lowest concentration causing 100% growth inhibition. For FCZ, VRZ, and ITZ, the MICs were the lowest concentrations that produced a 50% reduction in growth. However, for TRB, the MICs were defined as the lowest concentration that caused 80% inhibition of growth, compared to the drug-free growth control. The MICs that inhibited 50% (MIC_50_) and 90% (MIC_90_) of the isolates were calculated as previously described (Hamilton-Miller 1991).

### Chequerboard assay

To evaluate the drug interactions, a chequerboard microdilution test was conducted with three different combinations of antifungals: FCZ + AMB, FCZ + TRB, and AMB + TRB. The antifungals AMB, FCZ, and TRB were dissolved and diluted in RPMI 1640. The inoculums were 0.5 × 10^3^ to 2.5 × 10^3^ CFU/mL, and the drug dilutions ranged from 0.06 to 8 µg/mL for AMB, 0.25–16 µg/mL for FCZ, and 0.06–8 µg/mL for TRB. The plates were incubated at 35 °C for 72 h. The drug interaction coefficient was evaluated using the fractional inhibitory concentration (FIC) index, which was calculated by the following formula:

$$\mathbf F\mathbf I\mathbf C=(\mathbf M\mathbf I\mathbf C\mathbf A\boldsymbol\;\mathbf i\mathbf n\boldsymbol\;\mathbf c\mathbf o\mathbf m\mathbf b\mathbf i\mathbf n\mathbf a\mathbf t\mathbf i\mathbf o\mathbf n/\mathbf M\mathbf I\mathbf C\mathbf A)+(\mathbf M\mathbf I\mathbf C\mathbf B\boldsymbol\;\mathbf i\mathbf n\boldsymbol\;\mathbf c\mathbf o\mathbf m\mathbf b\mathbf i\mathbf n\mathbf a\mathbf t\mathbf i\mathbf o\mathbf n/\mathbf M\mathbf I\mathbf C\mathbf B)$$

The interaction between antifungals was categorized based on the FIC index. If the FIC index was ≤ 0.5, the interaction was classified as synergistic. If the FIC index was > 0.5 and ≤ 4, the interaction was classified as indifferent. Conversely, if the FIC index was > 4.0, the interaction was deemed as antagonistic (Odds, 2003).

Clinical breakpoints (CBPs) are unavailable for antifungal drugs, so epidemiological cutoff values (ECVs) were calculated to provide an early warning of isolates with reduced susceptibility to the tested drug (Dalhoff et al. 2009; Espinel-Ingroff et al. 2012a, b). The ECV is the highest MIC value that represents the upper limit of the distribution of MICs for wild-type (WT) isolates (without acquired drug resistance). The ECV is determined by analyzing a population of isolates from a specific species and selecting the value that represents the highest end of the distribution of MICs. The ECVs were set at ≥ 97.5% of the MIC value of the statistically modeled population (Clinical and Laboratory Standards Institute (CLSI) 2022). Isolates having an MIC value higher than the ECV is interpreted as non-wild-type (NWT).

As recommended in CLSI, M57S-Ed4 and previous studies (Espinel-Ingroff et al. 2012a, b), the ECVs of AMB was 0.5 µg/mL for *C. neoformans* VNI and *C. gattii* VGI and 1 µg/mL for *C. gattii* VGII. The ECV of FCZ was 8 µg/mL for *C. neoformans* VNI and *C. gattii* VGIII, 16 µg/mL for *C. gattii* VGI and VNIII, and 32 µg/mL for *C. gattii* VGII. The ECV of ITZ and VRZ was 0.25 µg/mL for *C. neoformans* VNI, and the ECV of VRZ was 0.25 µg/mL for VNIII. The ITZ ECV was 0.5 µg/mL for C*. gattii* VGI and VGIII and 1 µg/mL for VGII. The VRZ ECV was 0.5 µg/mL for C*. gattii* VGI and VGII. The ECV for TRB was 1 µg/ mL against *C. neoformans* VNI (Reichert‐Lima et al. 2016).

### Data analysis

Statistical analysis and data visualization were performed with R software (R Core Team, 2022; version 4.2.0). Hierarchical clustering analysis of M13-fingerprinting and *URA5* RFLP of *C. neoformans* and *C. gattii* was performed using the unweighted pair group method with arithmetic mean (UPGMA). Dendrograms were constructed based on M13-fingerprinting and RFLP analysis of the *URA5* gene, using the “factoextra” package. The “Complex heatmap” package (Gu et al. 2016) was used to construct the heatmap, whereas, the “corrplot” package (Wei et al. 2017) was used to assess the correlation between MICs of antifungal drugs against *C. neoformans* and *C. gattii*. Furthermore, the “psych” package (Revelle 2011) was used to calculate the MIC geometric means. The significant difference between the MICs of each antifungal against each genotype was determined using one-way analysis of variance. Multiple comparisons between the means were assessed at significance thresholds obtained from the Bonferroni correction. *P*-values less than 0.05 were considered to indicate statistical significance.

## Results

### Isolation and identification of *Cryptococcus* spp.

A total of 400 environmental samples were collected, 220 from birds and 180 from trees. *Cryptococcus* spp. were found in 58 (14.5%) of the samples, 44 (75.9%) of which were isolated from birds and 14 (24.1%) from trees (Table 1). A total of 120 pigeon droppings were collected, 23 (19.17%) of which tested positive for *Cryptococcus* spp. Similarly, 8 (16%) *Cryptococcus* isolates were isolated from 50 captive bird droppings, and 13 (26%) isolates were isolated from 50 zoo bird droppings. In addition, 130 *Eucalyptus* tree samples were collected from Cairo, Sharkia Governorate, and the Cairo-Alexandria Desert Road, and 9 *Cryptococcus* isolates (6.92%) were obtained from the leaves and woody trunks. Furthermore, out of the 50 olive tree samples examined, 5 (10%) isolates were recovered (Table 1).

**Table 1:** Frequency of *Cryptococcus* spp. isolated from bird droppings and tree samples

Source		No. of collected samples	*Cryptococcus* spp. positive (%)	*C. neoformans* positive (%)	Genotype of *C. neoformans* (%)			*C. gattii* positive (%)	Genotype of *C. gattii* (%)		
					**VNI**	**VNII**	**VNIII**		**VGI**	**VGII**	**VGIII**
**Birds**	**Pigeons**	120	23 (19.2)	23 (100)	17 (73.9)	5 (21.7)	1 (4.4)	–	–	–	–
	**Captive**	50	8 (16.0)	8 (100)	7 (87.5)	1 (12.5)	0 (0.0)	–	–	–	–
	**Zoo**	50	13 (26.0)	–	–	–	–	13 (100)	7 (53.8)	4 (30.8)	2 (15.4)
**Trees**	***Eucalyptus***	130	9 (6.9)	–	–	–	–	9 (100)	8 (88.9)	1 (11.1)	0 (0.0)
	**Olive**	50	5 (10.0)	–	–	–	–	5 (100)	4 (80.0)	0 (0.0)	1 (20.0)
**Total**		**400**	**58 (14.5)**	**31 (53.4)**	**24 (77.4)**	**6 (19.4)**	**1 (3.2)**	**27 (46.4)**	**19 (70.4)**	**5 (18.5)**	**3 (11.1)**

*Cryptococcus* species were identified based on their phenotype (macromorphological, micromorphological, biochemical characters) and molecular identification.

### Differentiation of *C. neoformans* and *C. gattii* isolates

All the isolates were subcultured on CDA media and observed for any color change. After five days of incubation, 24 isolates were recovered, including 6 from *Eucalyptus* tree samples, 5 from olive tree samples, 12 from zoo bird droppings, and one from pigeon dropping. These isolates produced brown mucoid colonies and were identified as *C. gattii*. The other isolates, which included 3 from *Eucalyptus* tree samples, 22 from pigeon droppings, 8 from captive bird droppings, and one from zoo bird drooping, produced light blue dry colonies and were identified as *C. neoformans*.

Notably, one *C. gattii* isolate was identified as *C. neoformans* and four *C. neoformans* isolates were identified as *C. gattii* by multiplex PCR. Multiplex PCR was performed using the CNa-70S and CNa-70A primers for *C. neoformans* and the CNb-49S and CNb-49A primers for *C. gattii*, resulting in amplicons of 695 and 448 bp for *C. neoformans* and *C. gattii,* respectively. Of the 58 *Cryptococcus* spp. isolated, 31 (53.4%) were *C. neoformans* and 27 (46.4%) were *C. gattii* (Table 1). Both *C. neoformans* and *C. gattii* were isolated from birds, but *C. gattii* was found only on trees.

### Genotyping of *C. neoformans and C. gattii* species complexes

Fifty-eight *C. neoformans* and *C. gattii* isolates formerly identified using phenotypic methods and multiplex PCR were subjected to PCR fingerprinting with the M13 primer. PCR fingerprinting types VNI-VNIII and VGI-VGIII were assigned according to the typical major bands observed for each pattern (Supplementary Fig. 1A and B). Only visible bands were included in the analysis regardless of their intensity. Of the 31 *C. neoformans* isolates, 24 (77.4%), 6 (19.4%) and one (4.4%) belonged to the VNI, VNII, and VNIII genotypes, respectively (Table 1). Genotype VNIII was detected only in pigeon droppings. In contrast, the 27 *C. gattii* isolates belonged to VGI (70.4%), VGII (18.5%), and VGIII (11.1%) genotypes.

**Fig. 1:**
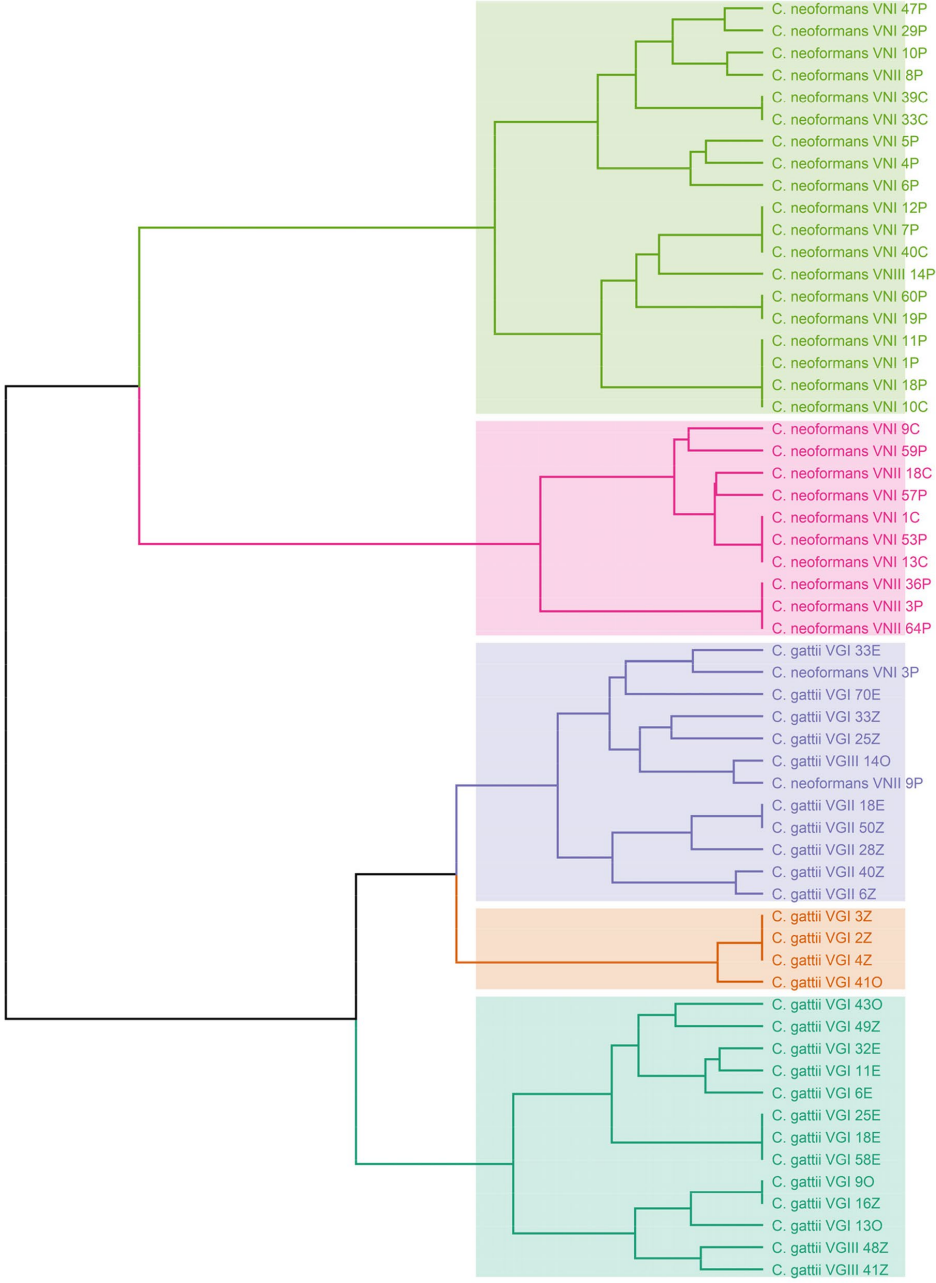
M13 PCR-fingerprinting and PCR–RFLP dendrogram of the *URA5* gene of *C. neoformans* and *C. gattii* genotypes

RFLP analysis of the *URA5* gene with the restriction enzymes *Sau961* and *HhaI* in a double digest, revealed two restriction patterns of 447 and 248 bp specific for *C. neoformans* and 324 and 124 bp for *C. gattii* (Supplementary Fig. 1C)*.* RFLP patterns were assigned visually by comparison with the patterns obtained from the standard strains (VNI-VNIII and VGI-VGIII). The RFLP analysis of the *URA*5 gene in *C. neoformans* and *C. gattii* revealed 28 unique profiles. A dendrogram was created to group strains based on their similarity, resulting in 5 clusters (Fig. 1). The first two clusters included *C. neoformans* from various sources, while the remaining three clusters included *C. gattii*. The genotypes of both *C. neoformans* and *C. gattii* were randomly distributed among the clusters.

### Antifungal susceptibility profiles

**Fig. 2:**
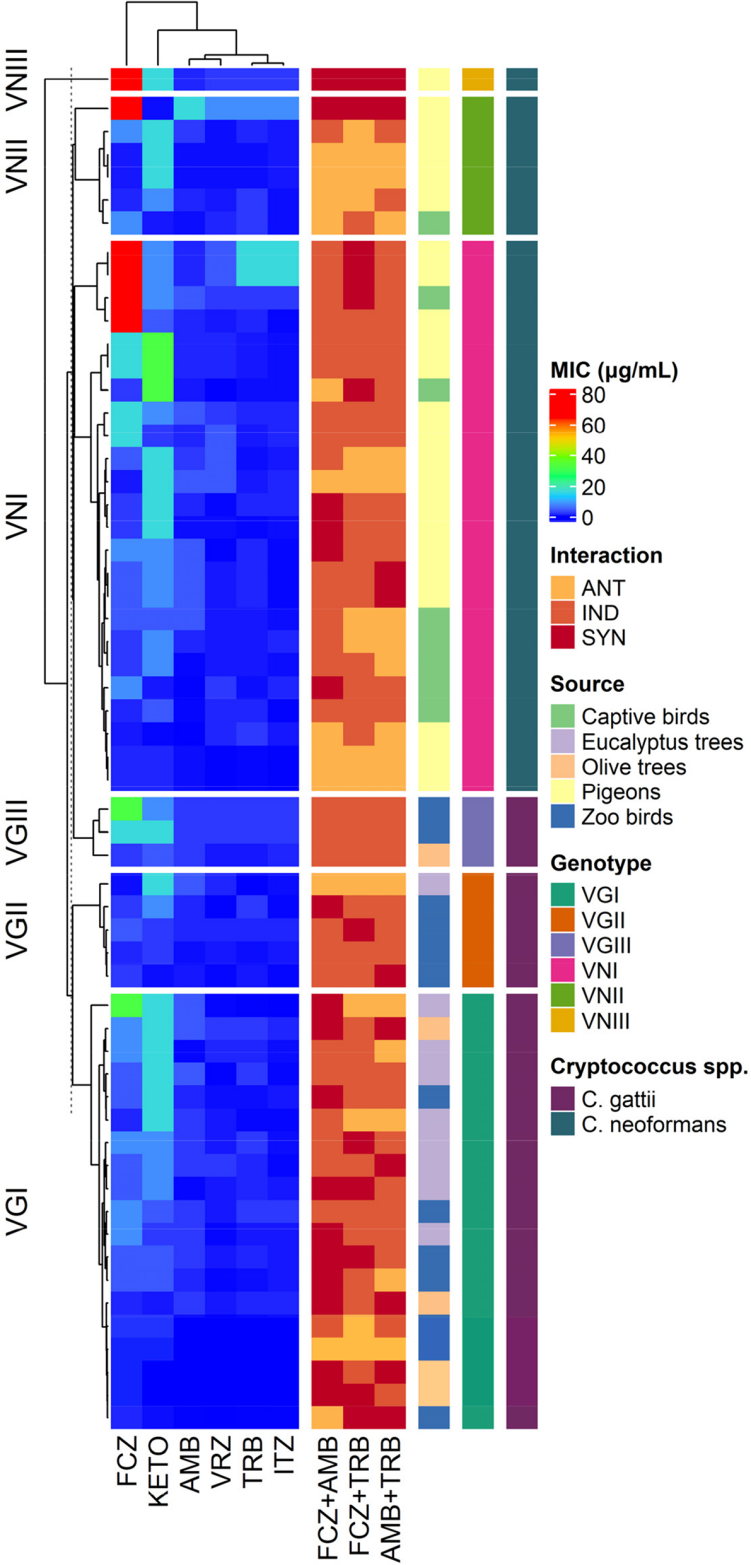
Heatmap representation of *Cryptococcus* spp. genotypes isolated from bird droppings and trees in Egypt, minimum inhibitory concentration (MIC), and interaction of antifungal agents

The antifungal susceptibility patterns of 31 *C. neoformans* and 27 *C. gattii* isolates are presented based on the genotype of each isolate (Fig. 2). Table 2 shows the MIC distributions for the tested antifungals against *C. neoformans* and *C. gattii* genotypes. Eight (33.3%) *C. neoformans* VNI isolates, along with one (16.7%) VNII and one (100%) VNIII isolate were NWT with reduced susceptibility to FCZ (MICs: 16 and 64 μg/mL). Similarly, three (11.1%) *C. gattii* isolates (one VGI and two VGIII isolates) have acquired resistance to FCZ (Table 2). Furthermore, 11 (45.8%) *C. neoformans* VNI isolates and six (22.2%) *C. gattii* (three VGI and three VGIII) were found to be NWT with possibly acquired ITZ resistance. Nineteen (61.3%) *C. neoformans* (18 VNI and one VNIII) isolates were observed to be NWT to VRZ. Also, five (18.5%) *C. gattii*, including three VGI and two VGII isolates had VRZ MIC exceeding ECVs. *C. neoformans* VNII isolate and *C. gattii* VGIII were also found to be NWT having MIC values of 8 μg/mL and 2 μg/mL, respectively. Sixteen (66.7%) *C. neoformans* VNI (MIC 1–4 µg/mL), twelve (63.2%) *C. gattii* VGI (MIC 1–8 µg/mL) and one (20%) *C. gattii* VGII (MIC 4 µg/mL) were NWT with possibly acquired AMB resistance. Eight (25.8%) *C. neoformans* isolates (seven VNI and one VNII) and six (22.2%) *C. gattii* isolates (five VGI and one VGII) were observed to have TRB MIC value of 1 μg/mL; thus 15 isolates including 7 (22.6%) *C. neoformans*, 5 (18.5%) *C. gattii* with an MIC of 2 μg/mL, two *C. neoformans* VNI (MIC = 16 μg/mL), and one VNII (MIC = 8 μg/mL) isolates may have acquired resistance to TRB.

**Table 2:**
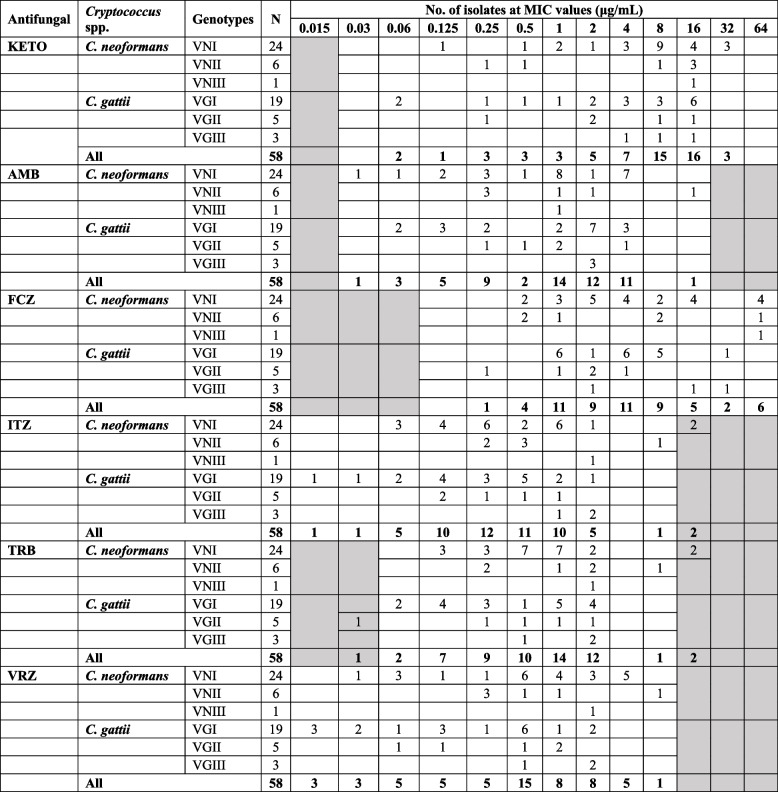
Distribution of MICs of antifungal drugs against *C. neoformans* and *C. gattii* genotypes

*KETO* Ketoconazole, *AMB* Amphotericin B, *FCZ* Fluconazole, *ITZ* Itraconazole, *TRB* Terbinafine, *VRZ* Voriconazole, *MIC* Minimum inhibitory concentration. Values in the grey cells indicate that the MICs lies outside the dilution range tested

The range, mode, MIC_50_, MIC_90_, and GMs for antifungal drugs tested are presented in Table 3. The MIC_90_ and susceptibility ranges of *C. neoformans* and *C. gattii* genotypes were 16 μg/mL (0.125–32; 0.062–16) for KETO, 4 μg/mL (0.03–16; 0.06–16 μg/mL) for AMB, 64;8 (0.5–64; 0.25–32 μg/mL) for FCZ, 2;1 (0.06–16; 0.015–2 μg/mL) for ITZ, 2 (0.125–16; 0.03–2 μg/mL) for TRB, and 4;1 (0.03–8; 0.015–2 μg/mL) for VRZ, respectively. MICs for the QC strain ATCC 22019 were within the CLSI established limits (FCZ: 2 μg/mL, AMB: 0.5 μg/mL, ITZ: 0.125 μg/mL, VRZ:0.06 μg/mL, and KETO: 0.25 μg/mL).

**Table 3:** MIC range, mode, MIC_50_, MIC_90_, and geometric means of antifungal drugs against *C. neoformans* and *C. gattii* genotypes

**Antifungal**	**Species**	**Genotype**	**N**					
				**Range**	**Mode**	**MIC**_**50**_	**MIC**_**90**_	**GM**
**KETO**	***C. neoformans***	VNI	24	0.125–32	8	8	32	5.82
		VNII	6	0.25–16	16	NA	NA	4.00
		All	30	0.125–32	8	8	16	5.40
	***C. gattii***	VGI	19	0.062–16	16	4	16	2.98
		VGII	5	0.25–16	2	NA	NA	2.64
		All	24	0.062–16	16	4	16	2.91
**AMB**	***C. neoformans***	VNI	24	0.03–4	1	1	4	0.81
		VNII	6	0.25–16	0.25	NA	NA	0.89
		All	30	0.03–16	1	1	4	0.83
	***C. gattii***	VGI	19	0.06–4	2	2	4	0.74
		VGII	5	0.25–4	1	NA	NA	0.87
		All	24	0.06–4	2	1	4	0.77
**FCZ**	***C. neoformans***	VNI	24	0.5–64	2	4	64	5.19
		VNII	6	0.5–64	8, 0.5	NA	NA	3.17
		All	30	0.5–64	2	4	64	4.70
	***C. gattii***	VGI	19	1–32	4 – 1	4	8	3.33
		VGII	5	0.25–4	2	NA	NA	1.32
		All	24	0.25–32	4, 1	4	8	2.75
**ITZ**	***C. neoformans***	VNI	24	0.06–16	1, 0.25	0.25	2	0.43
		VNII	6	0.25–8	0.5	NA	NA	0.63
		All	30	0.06–16	0.25	0.25	2	0.46
	***C. gattii***	VGI	19	0.015–2	0.5	0.25	1	0.22
		VGII	5	0.125–1	0.125	NA	NA	0.29
		All	24	0.015–2	0.5, 0.125	0.25	1	0.23
**TRB**	***C. neoformans***	VNI	24	0.125–16	0.5, 1	0.5	2	0.71
		VNII	6	0.25–8	2, 0.25	NA	NA	1.12
		All	30	0.125–16	1	0.5	2	0.78
	***C. gattii***	VGI	19	0.06–2	1	0.5	2	0.43
		VGII	5	0.03–2	NA	NA	NA	0.38
		All	24	0.03–2	1	0.5	2	0.42
**VRZ**	***C. neoformans***	VNI	24	0.03–4	0.5	0.5	4	0.64
		VNII	6	0.25–8	0.25	NA	NA	0.63
		All	30	0.03–8	0.5	0.5	4	0.64
	***C. gattii***	VGI	19	0.015–2	0.5	0.25	1	0.18
		VGII	5	0.06–1	1	NA	NA	0.33
		All	24	0.015–2	0.5	0.25	1	0.20

*MIC* Minimum inhibitory concentration, *ECV* Epidemiological cutoff value (MIC value at which ≥ 95% of the isolates are resistant), *GM* Geometric mean, *NA* Not available. MIC_50_ and MIC_90_ couldn’t be estimated for isolates less than 10

Multiple comparisons between MICs of *C. neoformans* and *C. gattii* genotypes showed no significant differences (*P* > 0.05) (Table 4). Similarly, no significant differences were found between MICs of *C. gattii* genotypes except between the MICs of FCZ and ITZ of genotypes VGI vs VGIII and VGII and VGIII. *C. gattii* VGIII showed less susceptibility to FCZ and ITZ than VGI and VGII (Table 4).

**Table 4:**
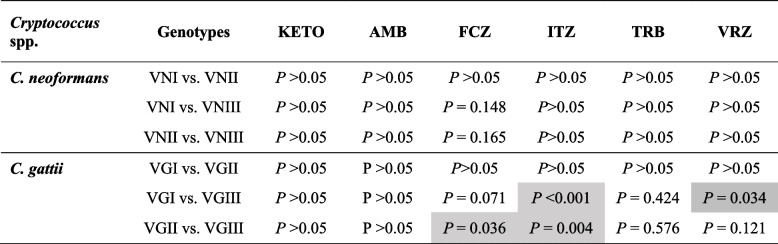
Comparison of MICs of antifungal agents against* C. neoformans* and *C. gattii* genotypes

Grey cells indicate significant at *P*-value < 0.05. *FCZ* Fluconazole, *AMB* Amphotericin B, *TRB* Terbinafine

**Fig. 3:**
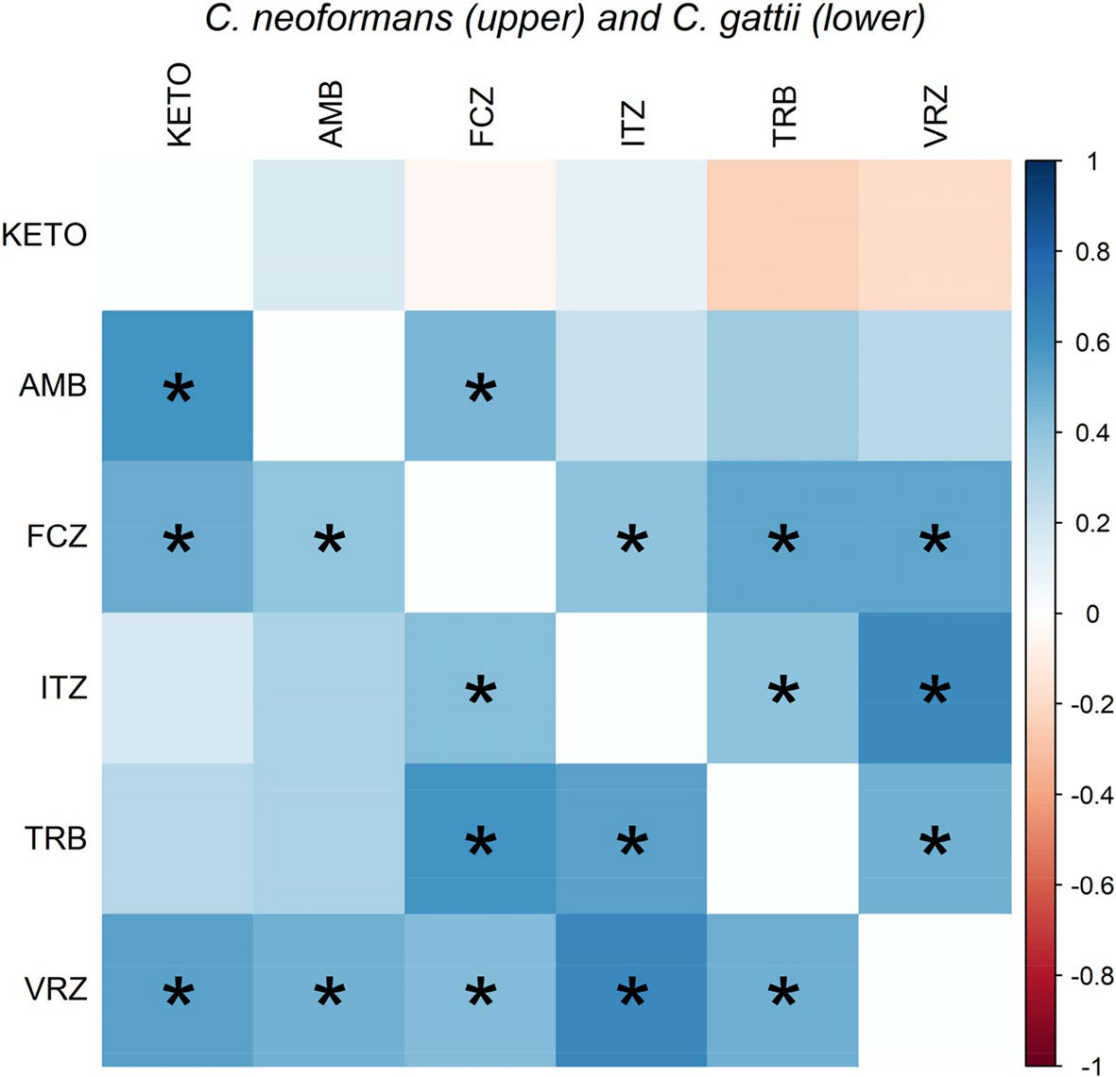
Spearman rank correlation test results based on the minimum inhibitory concentrations of antifungal agents against *C. neoformans* and *C. gattii* genotypes. Blue color indicated positive correlation and red show negative correlation. Strikes (*) indicates significant at *P* < 0.05

Many significant correlations were detected between MICs of antifungals against *C. neoformans* and *C. gattii* genotypes (Fig. 3). Spearman correlation analysis among MICs revealed significant (*P* < 0.001) correlations between VRZ and ITZ (*r* = 0.64) for both *C*. *neoformans* and *C. gattii* isolates; between FCZ and TRB for the *C. neoformans* isolates; and between FCZ and TRB (*r* = 0.52) for *C. gattii* isolates.

### Antifungal combinations for *C. neoformans and C. gattii* species complexes

The effects of three different antifungal combinations were tested against *C. neoformans* and *C. gattii* genotypes and the MICs before and after combination were evaluated (Table 5). When FCZ + AMB, FCZ + TRB and AMB + TRB were combined, the MICs of FCZ in combination were significantly lower than those of FCZ alone against both *C. neoformans* (*P* = 0.007 and 0.008) and *C. gattii* (*P* = 0.023 and 0.011) genotypes. However, the MICs of AMB in combination were significantly lower than those of AMB alone against only *C. gattii* (*P* = 0.015 and 0.003) genotypes. On the other hand, there is no significant differences in the MICs of TRB in combination with FCZ (*P* = 0.064) or in combination with AMB (*P* = 0.543) and that of TRB alone against *C. gattii* genotypes.

**Table 5:** Fractional minimum inhibitory concentration index of antifungal drug combinations against *C. neoformans* and *C. gattii* genotypes

Antifungal Combination	Antifungal	MIC (μg/mL) range of *C. neoformans*				MIC (μg/mL) range of *C. gattii*			
		**VNI** **(*n* = 24)**	**VNII** **(*n* = 6)**	**All** **(*n* = 30)**	***P*-value**	**VGI** **(*n* = 19)**	**VGII** **(*n* = 5)**	**All** **(*n* = 24)**	***P*-value**
**FCZ + AMB**	FCZ	0.5 – 64	0.5 – 64	0.5 – 64	0.007	1 – 32	0.25 – 4	0.25 – 32	0.023
	FCZ comb	0.125 – 8	1 – 8	0.125 – 8		0.031 – 8	0.5 – 2	0.031 – 8	
	AMB	0.03 – 4	0.25 – 16	0.03 – 16	0.396	0.06 – 4	0.25 – 4	0.06 – 4	0.015
	AMB comb	0.06 – 4	0.5 – 4	0.06 – 1		0.0156 – 4	0.25 – 1	0.0156 – 4	
**FCZ + TRB**	FCZ	0.5 – 64	0.5 – 64	0.5 – 64	0.008	1 – 32	0.25 – 4	0.25 – 32	0.011
	FCZ comb	0.125 – 8	4 – 8	0.125 – 4		0.03 – 4	0.5 – 2	0.03 – 4	
	TRB	0.125 – 16	0.25 – 8	0.125 – 16	0.343	0.06 – 2	0.03 – 2	0.03 – 2	0.064
	TRB comb	0.06 – 4	2 – 8	0.06 – 8		0.03 – 4	0.25 – 2	0.03 – 4	
**AMB + TRB**	AMB	0.03 – 4	0.25 – 16	0.03 – 16	0.226	0.06 – 4	0.25 – 4	0.06 – 4	0.003
	AMB comb	0.125 – 4	0.5 – 2	0.125 – 4		0.015 – 2	0.06 – 0.5	0.015 – 2	
	TRB	0.125 – 16	0.25 – 8	0.125 – 16	0.593	0.06 – 2	0.03 – 2	0.03 – 2	0.543
	TRB comb	0.125 – 8	1 – 4	0.125 – 8		0.015 – 2	0.06 – 0.5	0.015 – 2	

*MIC* Minimum inhibitory concentration, *FCZ* Fluconazole, *AMB* Amphotericin B, *TRB* Terbinafine, *Comb* in combination; *P*-value: of Paired *t*-test (significance level at *P*-value < 0.05)

When FCZ + AMB, FCZ + TRB, and AMB + TRB were combined, there was a synergistic effect against *C. neoformans* VNI of 16.7%, 16.7%, and 8.3%, respectively, and a synergistic effect against *C. neoformans* VNII of 16.7% for each combination. However, these combinations had synergistic effects against *C. gattii* VGI and VGII (52.6%, 26.3%, and 26.3%) and (20%, 20%, and 20%), respectively. Among all combinations, there was a 100% synergistic effect against *C. neoformans* VNIII and a 100% indifferent effect against *C. gattii* VGIII.

## Discussion

Yeast of the genus *Cryptococcus* is a highly potential basidiomycetous fungal pathogen for human and animal health. Inhalation of infective basidiospores is the primary route of infection with this fungus, and the environment plays a significant role in the spread of *C. neoformans* infection in humans and animals (May et al. 2016). *C. neoformans* and *C. gattii* species complexes are the global isolates responsible for *Cryptococcus* infection and are commonly recovered from pigeon droppings, soil, and decaying wood in hollow trees (Firacative et al. 2021). This study aimed to investigate the presence of *C*. *neoformans* and *C. gattii* species complexes isolates from environmental sources, and to determine their molecular types and antifungal susceptibility patterns. Notably, this study is the first to conduct genotyping and antifungal susceptibility analysis of environmental *C. neoformans* and *C. gattii* species complexes in Egypt.

Out of 400 environmental samples, 58 isolates (14.5%) were identified as *Cryptococcus* spp. This proportion is higher than that found in the study of Gugnani et al. (2020) in the Dutch Caribbean, where only 4.3% of the total isolates were identified as *Cryptococcus* spp. and were found in pigeon droppings and woody debris from various trees. Similarly, Chen et al. (2021) reported 61 (6.6%) *C. neoformans* isolates out of 929 pigeon droppings in East China. However, this study did not isolate any *C. neoformans* or *C. gattii* from 309 samples of decayed debris from tree hollows (Chen et al. 2021).

This study revealed that *C. neoformans* was present in both pigeon and captive bird droppings, with recovery rates of 19.17% (23/120) and 16% (8/50), respectively. These results are consistent with the findings of Sirag et al. (2021) and Dou et al. (2017), who reported that *C. neoformans* was found in 16.67% and 18.6% of pigeon excreta in Mekkah, Saudi Arabia and China, respectively. In contrast, lower isolation rates were reported in India (11.6% from tree debris and 3.3% from avian excreta) and Thailand (11%) from pigeon droppings (Krangvichain et al. 2016; Prakash et al. 2018). Furthermore, lower isolation rates were recorded in the Brazilian Amazon, where *C. neoformans* was recovered from pigeon (4.7%) and captive bird (5%) droppings (Alves et al. 2016). Another study reported a lower detection rate (7.5%) in Egypt from caged bird excreta (Elhariri et al. 2015), while a higher percentages of *C. neoformans* were isolated from pigeon droppings in Saudi Arabia (32%) (Abulreesh et al. 2015), Libya (34%) (Ellabib et al. 2016), and southeastern Nigeria (22%) (Nweze et al. 2015).

Our study showed that *C. gattii* was present in the leaves and woody trunks of olive trees (10%) and *Eucalyptus* trees (6.92%), indicating that these trees are crucial reservoirs for *Cryptococcus* spp. The isolation rates from *Eucalyptus* trees were slightly lower than the 11.8% reported in southern Italy (Romeo et al. 2011) and 12% in Nairobi, Kenya (Kangogo et al. 2014). However, in Croatia, Pllana-Hajdari et al. (2019) reported a lower isolation rate of *C. neoformans* (0.8%) from olive trees and other tree species and (0%) from bird excreta. On the other hand, in Turkey, Ergin et al. (2019) found *C. gattii* in 22.4% of olive trees and 24.2% of *Eucalyptus* tree trunks. Differences in the isolation rates of *C. neoformans* and *C. gattii* species complexes may be attributed to intrinsic differences in colonization rate, isolation protocols, sample quality, the period of the study, other environmental factors in certain geographical regions, and methodological approaches carried out by researchers (Gutch et al. 2015).

Among the 58 genotyped environmental isolates of *C. neoformans* and *C. gattii* species complexes in the present study, C. *neoformans* VNI was the most frequent genotype (41.38%). Similarly, the molecular type VNI was the common genotype among environmental isolates recovered from East China (Chen et al. 2021) and the northern and southern Italy (Pini et al. 2017). Park et al. (2015) and Firacative et al. (2021) also reported that VNI was the most common molecular type among clinical and environmental isolates in Korea and Latin America. However, our findings differed from those of Dou et al. (2017) who reported a lower rate of *C. neoformans* VNI (18.6%) in China.

Clinical breakpoints (CBPs) for the C*. neoformans* and C*. gattii* species complexes are unavailable because they rely on pharmacokinetic and pharmacodynamic parameters, animal studies, and clinical outcomes of therapy (Espinel-Ingroff et al. 2012b). Additionally, there are limited data on the ECV of environmental *C. neoformans* isolates (Espinel-Ingroff et al. 2012a, b). The absence of recognized cutoff points for interpreting antifungal susceptibility results makes it difficult to characterize *Cryptococcus* spp. antifungal resistance in the laboratory. ECVs offer a sensitive mean to identify evolving antimicrobial resistance when CBPs are absent (Pfaller et al. 2011). However, when each genotype was assessed independently, the ECV values varied, indicating that the levels of ECVs may differ depending on the genotype and/or species involved (Reichert-Lima et al. 2016).

*Cryptococcus* isolates in the environment may reach humans through various transmission pathways. Therefore, the antifungal susceptibility profiles of these isolates can aid in the development of treatment guidelines. *C. neoformans* isolates have increasingly shown FCZ-resistance, varying by strain genotype and geographical location (Espinel-Ingroff et al. 2012a, b; Gullo et al. 2013; Fan et al. 2016). The main prescribed antifungal drug for treating cryptococcosis is AMB combined with FCZ and/or 5-FC, with occasional use of ITC and other azoles (Perfect et al. 2010). In this study, the susceptibility of 58 environmental isolates to six antifungal compounds (AMB, KETO, FCZ, ITZ, VRZ, and TRB) was assessed. NWT isolates with possibly acquired AMB resistance was found in 29/58 (50%) of the tested isolates including 16 *C. neoformans* VNI (MIC 1–4 µg/mL), twelve *C. gattii* VGI (MIC 1–8 µg/mL), and one *C. gattii* VGII (MIC 4 µg/mL). Similarly, Guerra et al. (2012) and Andrade-Silva et al. (2013) reported an increasing number of AMB- acquired resistance (MIC ≥ 2 µg/mL) in clinical *C. neoformans* (12/20, 60%) and (10% of 95) isolates, respectively. However, Reichert‐Lima et al. (2016) found *C. neoformans* VNI, VNII, and *C. gattii* VGII clinical isolates were WT to AMB (MIC ≤ 1 µg/mL).

Thirteen *C. neoformans* and *C. gattii* isolates (22.4%) had FCZ MICs exceeding the ECVs (MIC = 16–64 µg/mL; *C. neoformans* MIC_90_ was 64 µg/mL and that of *C. gattii* was 8 µg/mL). Seventeen isolates (18.97%) including 11 *C. neoformans* VNI (0.5–16 µg/mL; MIC_90_ 2 µg/mL) and 6 *C. gattii* isolates (1–2 µg/mL; MIC_90_ 1 µg/mL) can be categorized as ITZ NWT. A higher ITZ NWT environmental isolates (41%; MIC: 0.5–1 µg/mL) was reported in China (Chen et al. 2021) along with one FCZ NWT isolate (32 µg/mL). Gutch et al. (2015) reported that 8.6% of Indian environmental *C. neoformans* isolates (MIC_90_ 32 µg/mL) and 40% of *C. gattii* (MIC_90_ 64 µg/mL) were FCZ NWT, with lower ITZ NWT *C. neoformans* isolates (5.2%; MIC_90_ 0.5 µg/mL), and WT *C. gattii*. A much lower FCZ and ITZ MIC_90_ (4 µg/mL and 0.094 µg/mL) for *C. neoformans* isolates from decayed trunks of hollows were found in northwestern India (Khan et al. 2007). Chowdhary et al. (2011) reported increased susceptibility of environmental *C*. *grubii* and *C. gattii* isolates to FCZ (MIC_90_ 4 µg/mL and 8 µg/mL), ITZ (MIC_90_ 0.250 µg/mL and 0.5 µg/mL), VRZ (MIC_90_ 0.125 µg/mL and 0.250 µg/mL), and AMB (MIC_90_ 0.250 µg/mL), respectively. Moreover, Gutch et al. (2015) and Khan et al. (2007) observed higher susceptibility of *C. neoformans* and *C. gattii* isolates to KETO (MIC_90_ 0.064 µg/mL) vs. those reported in our study (MIC_90_ 16 μg/mL) for *C. neoformans* and *C. gattii* isolates. Guerra et al. (2012) reported that all *C. neoformans* clinical isolates, including AMB and ITZ NWT, were WT to TRB with MIC_50_ < 0.5 µg/mL, but our study showed 15/58 (25.86%) of isolates were TRB NWT, including 7 *C. neoformans*, 5 *C. gattii* having MIC 2 μg/mL, two *C. neoformans* VNI (MIC = 16 μg/mL) and one VNII (MIC = 8 μg/mL). Although KETO and TRB have shown in vitro activity against *C. neoformans/ C. gattii* clinical isolates, they are ineffective and toxic in the treatment of cryptococcal meningitis and should not be recommended Ghannoum and Rice 1999). Most clinicians would never consider the use of KETO in humans due to its inherent toxicities, and it would never be considered for use if any other azole was available. Currently, there is no available data on the use of TRB in central nervous system (CNS) fungal infections. Moreover, it is inconceivable that orally administrated TRB could effectively cross the blood–brain barrier and decrease the *Cryptococcus* burden in a CNS infection (Sorensen et al. 2000). TRB may be indicated for non-pulmonary, non-CNS cryptococcosis cases (such as cutaneous or intestinal cryptococcosis) but should not be indicated in other situations (Olsen et al. 2012). Despite reports of lower VRZ NWT clinical isolates (Pfaller et al. 2011; Reichert-Lima et al. 2016) or undetectable acquired resistance in clinical and environmental *Cryptococcus* isolates (Andrade-Silva et al. 2013), 44.83% of our environmental isolates were considered to be NWT to VRZ (MIC values of 2—8 μg/mL). Similarly, NWT *C. neoformans* VNI and *C. gattii* VGI, VGII, and VGIII isolates that may have acquired resistance to ITZ, FCZ, and VRZ have been reported in Brazil and Latin America (Silva et al. 2012; Brito-Santos et al. 2019; Grizante Barião et al. 2020; Firacative et al. 2021).

Considering that the susceptibilities of *C. neoformans* (VNI and VNII) and *C. gattii* (VGI and VGII) isolates differed, *C. neoformans* isolates had higher MICs of FCZ, ITZ, and VRZ than *C. gattii* (Table 4). Both species have nearly similar susceptibilities to KETO, AMB, and TRB (MIC_90_ 16 µg/mL_,_ 4 µg/mL, and 2 µg/mL, respectively) (Tables 3 and 4). However, previous studies have reported contrasting results. Gutch et al. (2015) found that *C. gattii* isolates may have acquired resistance to FCZ and KETO than *C. neoformans* isolates, and Reichert-Lima et al. (2016) found significant differences between the susceptibility of C. *gattii* VGII and *C. neoformans* VNI to FCZ, ITZ, and TRB, with the *C*. *gattii* VGII being NWT. Additionally, *C. gattii* VGIII was less susceptible to FCZ and ITZ than VGI and VGII (Tables 3 and 4). Trilles et al. (2012) reported that *C*. *gattii* VGII was the least WT to the tested antifungals followed by *C. neoformans* VNI and *C. gattii* VGI, indicating a relationship between genotype and antifungal susceptibility profile.

Low MICs were observed for the investigated combinations (Table 5), and a synergistic effect against *Cryptococcus* was noted, indicating that fewer drug doses are needed when antifungal agents are combined (Reichert-Lima et al. 2016). Other studies have reported a similar in vitro synergistic effects between AMB + FCZ, AMB + TRB and TRB + FCZ against clinical *C. neoformans* VNI, VNII, and *C. gattii* VGII (Reichert‐Lima et al. 2016); between azoles and TRB against *Pythium insidiosum* (Argenta et al. 2008), *Cladophialophora carrioni*, *Fonsecaea pedrosoi*, *Phialophora verrucosa* (Yu et al. 2008), and *Mucor irregularis* (Zhang et al. 2013). Guerra et al. (2012) reported a synergistic antifungal effect of TRB when TRB was combined with AMB, FLC, or ITC. The antifungal drugs used in the TRB + AMB and FCZ + TRB combinations inhibited or disrupted the ergosterol in the cell membrane through various mechanisms may explain the synergistic effects of these compounds (Zhang et al. 2013). Olsen et al. (2012) reported intestinal cryptococcosis caused by *C. neoformans* sensu lato in a dog that was unsuccessfully treated with AMB and FCZ. However, with TRB treatment, the case exhibited a full remission of clinical symptoms and a decrease in the cryptococcal antigen titre.

## Conclusions

In conclusion, our study demonstrated that VNI and VGI are the dominant genotypes of C*. neoformans* and C*. gattii* species complexes among environmental isolates in Egypt. Notably, this study also detected NWT isolates that may have acquired azole-resistance such as FCZ, ITR, and VRZ, and TRB- resistance. The FCZ and AMB combination demonstrated synergistic effect against the tested genotypes. To develop new therapeutic approaches for treating cryptococcosis, further investigations combining various antifungal drugs in vitro and in vivo are needed.

### Supplementary Information

Supplementary Material 1.

## Data Availability

Data supporting the findings of this study are available within the article and its supplementary materials.

## Abbreviations

PCR–RFLP: Restricted fragment length polymorphism
MICs: Minimum inhibitory concentrations
FIC index: Fractional inhibitory concentration index
ECVs: Epidemiological cutoff values
WT: Wild-type
NWT: Non -wild-type
AMB: Amphotericin B
TRB: Terbinafine
KETO: Ketoconazole
FCZ: Fluconazole
ITZ: Itraconazole
VRZ: Voriconazole
