# Supplementary figures and images for: Genetic diversity and antifungal susceptibilities of environmental Cryptococcus neoformans and Cryptococcus gattii species complexes
Source: IMA Fungus. 2024 Jul 25;15:21. doi: 10.1186/s43008-024-00153-w (PMC11282759; doi:10.1186/s43008-024-00153-w)

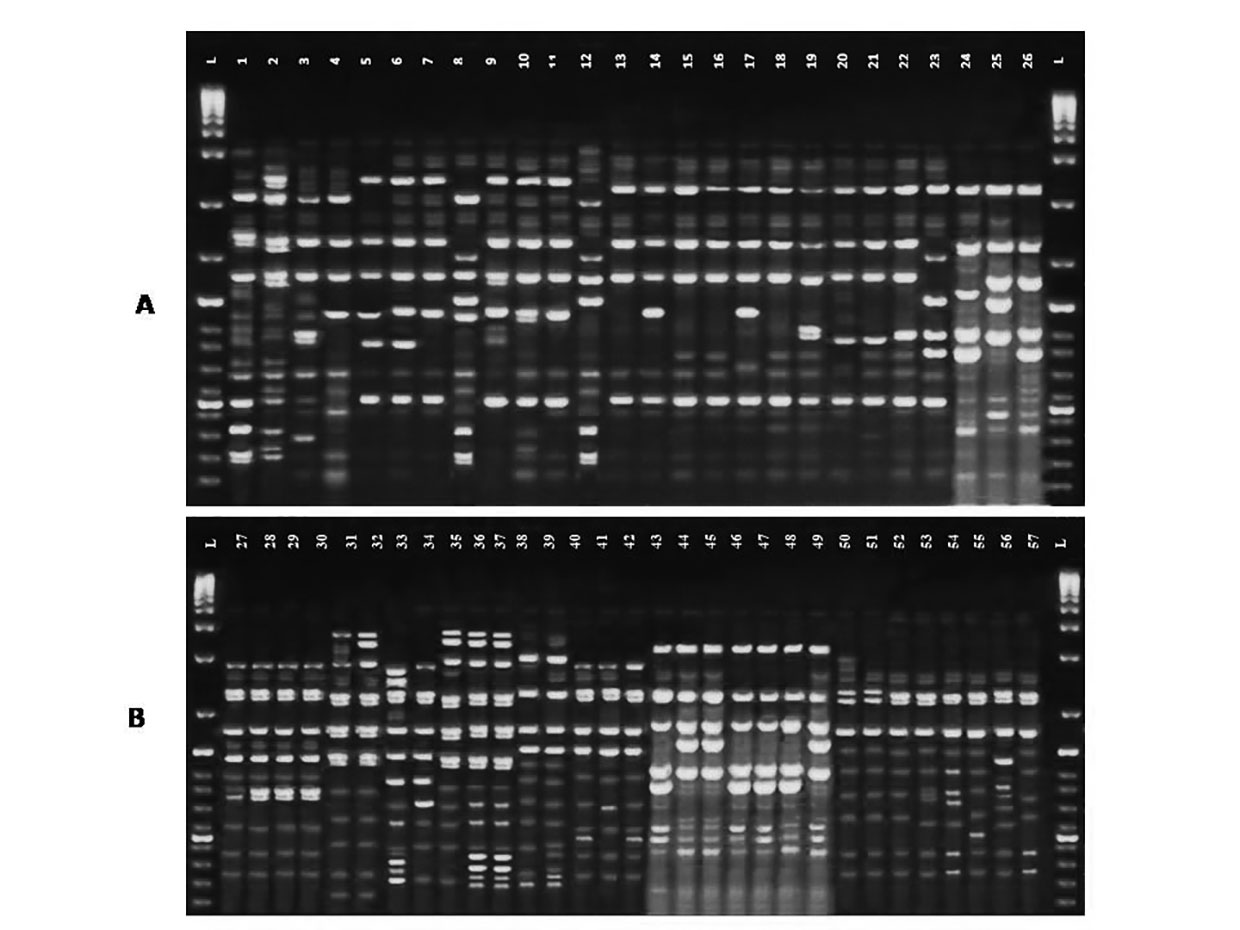

Supplement: Supplementary file 1 — Supplementary Material 1. [file 43008_2024_153_MOESM1_ESM.zip › Suppl Fig 1A,B.jpg]

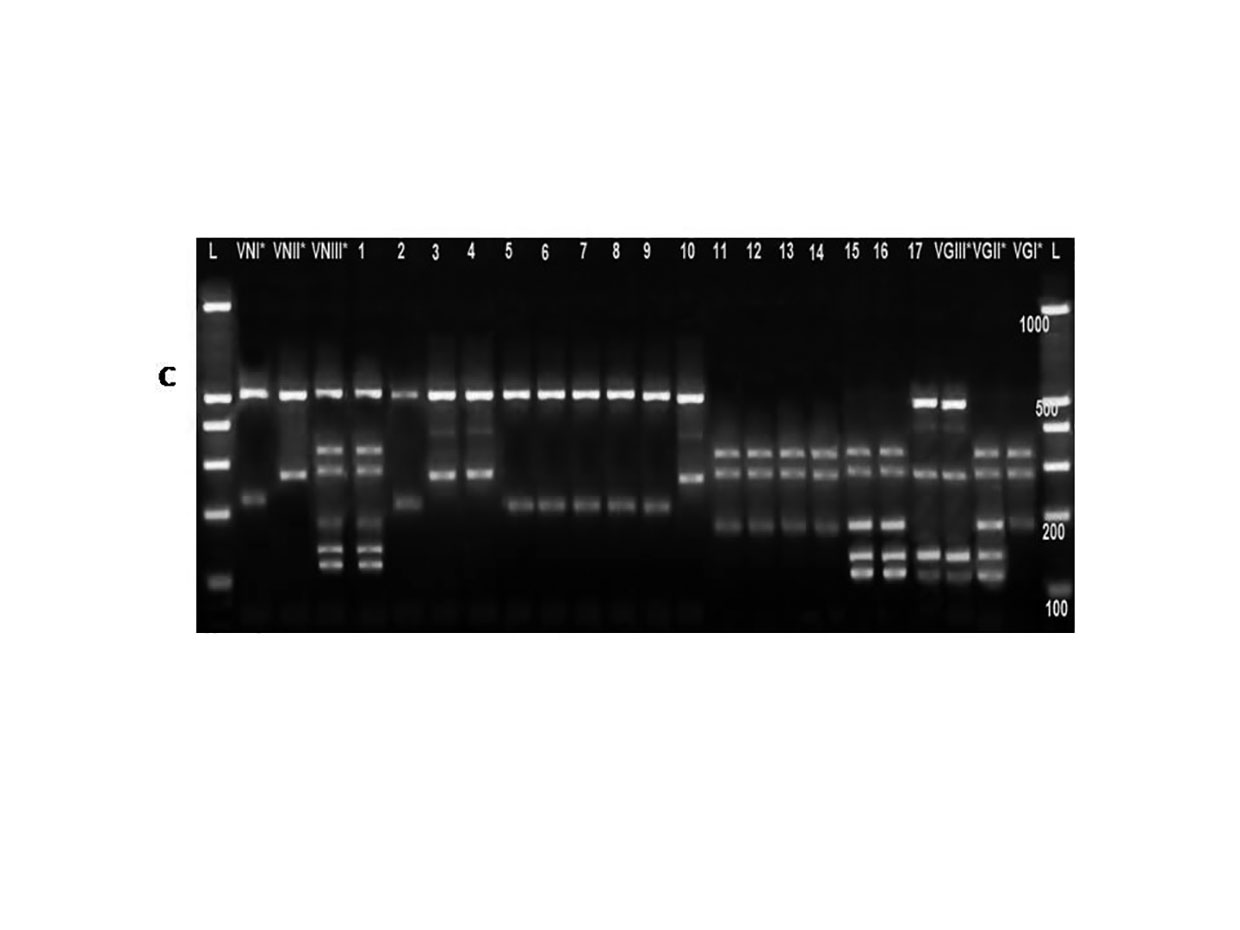

Supplement: Supplementary file 1 — Supplementary Material 1. [file 43008_2024_153_MOESM1_ESM.zip › Suppl Fig 1C.jpg]
